# MEMS Pressure Sensors with Novel TSV Design for Extreme Temperature Environments †

**DOI:** 10.3390/s25030636

**Published:** 2025-01-22

**Authors:** Muhannad Ghanam, Peter Woias, Frank Goldschmidtböing

**Affiliations:** Laboratory for Design of Microsystems, IMTEK—University of Freiburg, 79110 Freiburg im Breisgau, Germany; woias@imtek.de (P.W.); frank.goldschmidtboeing@imtek.uni-freiburg.de (F.G.)

**Keywords:** absolute sensor, diffusion bonding, eutectic bonding, Through-Silicon Vias, thermal drift

## Abstract

This study introduces a manufacturing process based on industrial MEMS technology, enabling the production of diverse sensor designs customized for a wide range of absolute pressure measurements. Using monocrystalline silicon as the structural material minimizes thermal stresses and eliminates temperature-dependent semiconductor effects, as silicon functions solely as a mechanical material. Integrating a eutectic bonding process in the sensor fabrication allows for a reliable operation at temperatures up to 350 °C. The capacitive sensor electrodes are enclosed within a silicon-based Faraday cage, ensuring effective shielding against external electrostatic interference. An innovative Through-Silicon Via (TSV) design, sealed using gold–gold (Au-Au) diffusion and gold–silicon (Au-Si) eutectic bonding, further enhances the mechanical and thermal stability of the sensors, even under high-temperature conditions. The unfilled TSV structure mitigates mechanical stress from thermal expansion. The sensors exhibited excellent performance, achieving a linearity of 99.994%, a thermal drift of −0.0164% FS (full scale)/K at full load and 350 °C, and a high sensitivity of 34 fF/kPa. These results highlight the potential of these sensors for high-performance applications across various demanding environments.

## 1. Introduction

Continuous monitoring and evaluation of process parameters in industry are crucial for controlling technical systems and for the early detection and prevention of errors and potential failures. Pressure and force sensors play a key role in this regard [1]. Despite being an established area of microsystem technology with mature products and high turnovers, there is still a need for research, especially in environments with extreme conditions such as high temperatures [2].

In many demanding applications, including environmental monitoring, industrial process control, aerospace, petrochemicals, and combustion process monitoring in power plants, sensors operate under extreme conditions [3,4]. Conventional sensor technologies often reach their limits and cannot fully meet specific requirements, such as higher operating temperatures, improved thermal and mechanical stability, and increased sensitivity [5,6]. Some of the key scientific and industrial requirements and development goals prioritized in [7] are as follows:

- Expansion of the usable pressure and temperature range;
- Increase in the long-term stability of the sensor signal;
- Improvement of media resistance;
- Reduction of the temperature dependence of the sensor signals.

These requirements are further underscored by the pursuit of cost reduction. While various technologies such as optical, piezoelectric, piezoresistive, and capacitive methods exist and offer a wide range of solution concepts, they cannot always fulfill all the above-mentioned requirements. Therefore, despite advanced developments in this field, the industry is constantly seeking new MEMS solutions that can meet these requirements.

This paper presents absolute pressure sensors designed for high-temperature applications. The innovative design and construction of these sensors address many of the limitations associated with conventional technologies. In earlier work, we briefly presented the initial development and results of absolute sensors operating up to 220 °C, enabled by the integration of innovative Through-Silicon Via (TSV) technology [8]. However, the information provided at that time was limited compared to the more comprehensive insights presented in this study. Here, we delve deeper into the advanced TSV fabrication process and sensor design.

To provide a clearer context for this work, the following section reviews conventional technologies and evaluates their ability to meet the aforementioned requirements. The third section focuses on the sensor design, supported by finite element analysis to determine the optimal design parameters, and provides a detailed description of the manufacturing process. The fourth section presents mechanical tests and measurements conducted at temperatures up to 350 °C, showcasing the sensors’ exceptional mechanical and thermal stability, high linearity, and long-term reliability. The final sections offer a comprehensive discussion and conclusion.

## 2. State of the Art

### 2.1. Widely Used Pressure Sensor Technologies

Due to the low manufacturing costs, long-term stability, high sensitivity, wide dynamic range, and high-temperature resistance, fiber optic measurement technology has developed rapidly in recent years [9]. Furthermore, optical strain gauges or fiber optic sensors (FOSs) offer the possibility of measuring various physical and biological variables, such as acceleration, strain, moisture, shape, temperature, pressure, corrosion, and pH value [10,11,12,13,14]. Unlike conventional electrical sensors, FOSs do not need to be supplied with electrical energy. This makes them completely passive and insensitive to electromagnetic interference (EMI) [1,15]. However, as optical fibers are inherently very sensitive to temperature, measuring slowly varying strain fields with temperature changes is a major challenge [11,14]. Typically, a temperature variation of 1 K can induce a strain equivalent to 10 µε. Consequently, when employing fiber optic sensors (FOSs), it becomes imperative to implement temperature compensation for accurate measurements.

Piezoelectric sensors operate by converting mechanical forces into analog voltage signals [16,17,18,19,20]. When subjected to mechanical loads, the piezoelectric crystal structure deforms and generates a change in the surface potential, which is directly proportional to the applied force. Due to this property, piezoelectric sensors do not require an external power source, as they are self-powered. These sensors are recognized for their high sensitivity, robust performance, and suitability across wide measurement ranges (typically spanning 0.7 kPa to 70 MPa) [21]. However, their signal yield, typically between 1 and 700 pC/N [22], is relatively low, necessitating the use of sophisticated electronic interfaces. To minimize signal loss caused by the high sensor impedance, the signal processing electronics must be placed in close proximity to the sensor, which complicates sensor integration, especially in high-temperature environments [21]. Moreover, piezoelectric sensors are unsuitable for static measurements, which limits their applications.

Piezoresistive sensors, the most widely utilized type of force and pressure sensors, employ resistive elements that are diffused, implanted, or deposited directly onto a stress-sensitive membrane [21,23]. The output signal correlates with the stress and strain applied to the membrane. Piezoresistive sensors are characterized by their high sensitivity, excellent linearity, and relatively straightforward signal processing requirements [24]. Nevertheless, their primary drawbacks include significant power consumption and output signal variability due to temperature dependence, which necessitates electronic compensation. As stress-sensitive devices, their output can also be influenced by mechanical or thermal stresses arising from mismatched coefficients of thermal expansion between the sensor and its housing material [25]. Furthermore, silicon-based piezoresistive sensors are constrained by leakage currents at insulating pn-junctions, which increase with temperature, limiting their operational range to approximately 150 °C [3]. Advanced materials such as silicon-on-insulator (SOI) or silicon carbide (SiC) enable sensor operation at temperatures up to 350 °C. However, these solutions incur significantly higher manufacturing costs and require more complex fabrication processes [26,27].

Compared to piezoresistive sensors, capacitive sensors exhibit a significantly lower temperature drift since their capacitance is typically not influenced by diffusion processes. Capacitive sensors are commonly designed as parallel-plate capacitors [1,23]. Pressure changes deform the sensitive membrane, altering the electrode gap or the dielectric properties (i.e., the relative dielectric constant *ε_r_*) of the medium between the plates, thereby affecting the capacitance. Capacitive pressure sensors are versatile, covering a wide pressure range from as low as 250 Pa to high pressures up to 70 MPa [21]. They offer several advantages, including high pressure sensitivity, excellent accuracy, low power consumption, and robust shock resistance [28,29]. However, challenges arise from parasitic and stray capacitances, which complicate capacitance measurements and the linearization of output signals [30]. Additionally, their fabrication often involves intricate packaging methods such as anodic bonding, silicon–silicon direct bonding (fusion), and glass frit bonding [31,32]. These bonding methods also present difficulties in establishing reliable electrical connections between the sensor electrodes and contact pads, which remains a significant challenge in the design and manufacturing of these sensors [31].

The main advantages and disadvantages can be summarized in Table 1.

### 2.2. Background on Through-Silicon Vias (TSVs)

**Figure 1:**
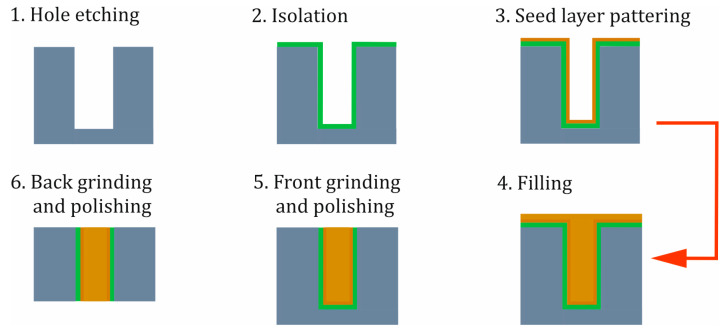
Standard process flow for the production of TSVs.

Absolute pressure sensors require a hermetically sealed cavity as a reference pressure chamber. In some cases, it is also necessary to protect the sensor from the measured medium. However, packaging is often a limiting factor, especially when it comes to transferring a product from design concepts to functional prototypes and finally to series production [34]. Anodic bonding, silicon–silicon direct bonding, and glass frit bonding are the most important methods for producing the sealed cavity. These packaging methods require complex equipment, high process temperatures, high voltage, and critical pretreatments [31,32]. Silicon stacking technologies or three-dimensionally integrated systems in a package (3D-SiP), which are based on the use of silicon TSVs, are another option [35]. In addition to creating a hermetically sealed cavity, TSVs offer the possibility of improving the performance, functionality, and density of integrated circuits (ICs) [36]. This enables the production of miniaturized, media-resistant sensors with higher temperature stability. However, this is usually associated with increased effort and costs. In general, TSVs are manufactured by etching trenches in silicon and then filling them sequentially with insulating and conductive materials [34,35,36,37,38] (Figure 1). The manufacturing process involves further critical process steps such as annealing to remove stress between the materials, high-temperature diffusion processes, grinding, and polishing.

These processes increase the complexity of production and are often expensive and time-consuming. In addition, process steps such as grinding and polishing are not always suitable for the microstructuring of silicon pressure sensors and can damage the sensitive membrane [27]. The hermetic seal of the TSV is achieved by filling the entire opening of the via with conductive material. The conductive material is usually copper, which has a low resistivity and high thermal conductivity [36]. However, there are concerns about the reliability of TSVs in high-temperature applications. It is known that thermal cycling methods, such as annealing, can lead to a mechanical failure of TSVs [39,40]. The different coefficients of thermal expansion of the filler material and the silicon wafer lead to significant mechanical stresses that impair the performance of the components. To optimize the performance of TSVs at high operating temperatures, various techniques such as thermal management and the reduction in electromigration can be used [41,42]. For example, thermal vias can be developed to transfer heat away from the TSVs and reduce problems such as thermal expansion and Cu extrusion at high temperatures. Another example is the application of barrier layers in TSV fabrication, such as tantalum nitride (TaN) or platinum (Pt), and passivation layers to minimize electromigration by restricting the movement of metal atoms due to electrical current flow. While these techniques improve reliability and functionality, they also increase the complexity of design and manufacturing.

A concept for manufacturing TSVs is presented in [34]. A trench is etched into a highly doped conductive silicon wafer and filled with insulating material. The advantage of this process is that the TSVs are suitable for high operating temperatures. Furthermore, voids, which are often caused by filling with conductive material, can be avoided. However, this process is only suitable for heavily doped wafers or SOI wafers. In addition, it is often not possible for medium-sized and small companies to carry out a via-first process in-house, and ordering wafers with predefined VIAs is often associated with long delivery times and high costs. Feng et al. [27] demonstrated an ultra-small pressure sensor with backside TSV contacts. The TSVs were created using a dry etching process, followed by multiple high-temperature depositions of SiO_2_ and doped polysilicon, which significantly increases the complexity and cost of fabrication.

This paper presents the proof-of-concept phase of high-temperature absolute pressure sensors with an innovative design of TSVs. In contrast to conventional TSVs, the via holes are completely open in this approach. Nevertheless, the final process leads to their hermetic sealing. In addition, the production of the TSVs is successfully integrated into the sensor manufacturing process without additional time-consuming steps and complex procedures such as grinding, polishing, high-temperature diffusion processes, or electroplating.

## 3. Materials and Methods

The challenge of this work lies in the fabrication of absolute pressure sensors suitable for high-temperature applications and harsh environments. The sensors are intended to stand out for their high thermal and mechanical stability, sensitivity, accuracy, long-term stability, and low energy consumption. To achieve this, the following design aspects based on existing technologies need to be considered:

- When using semiconductor devices, such as piezoresistive sensors, the measurement signal is significantly influenced by temperature-dependent semiconductor effects, which in turn can limit the operating temperature.
- Achieving low-noise readouts of the sensors is generally challenging due to the low signal yield, for example with piezoelectric sensors, or due to parasitic capacitances as in capacitive sensors.
- The use of SOI wafers and SiC substrates, as well as Si stacking technologies, provides a good opportunity for the production of high-temperature sensors, but increases process complexity and costs.
- The use of different materials in the sensor or package manufacturing leads to mechanical stresses due to the different temperature coefficients, which distort the output signal.
- The use of TSVs is advantageous for manufacturing miniaturized sensors and hermetically sealed reference chambers. However, TSVs have other drawbacks related to reliability and mechanical stress at higher operating temperatures. Additionally, most small and medium-sized enterprises rely on external suppliers for TSVs, which entails high costs and long lead times.
- Capacitive sensors, the majority of the presented sensors, operate with extremely thin membranes on small surfaces, resulting in low base capacitances. Furthermore, for high-temperature applications, two effects must be considered: the temperature-dependent changes in the dielectric constant of the dielectric material and the thermal stress at the interfaces between the various components of the sensor.

Furthermore, the manufacturing processes are largely design-dependent, and only one sensor design can be produced in a batch. This significantly affects the unit price for custom orders with small to medium batch sizes. Therefore, cost reduction in the manufacturing processes is always desirable.

### 3.1. Sensor Design

Considering the previous arguments, we have developed a concept for manufacturing cost-effective, miniaturized relative pressure and force sensors in previous works [33]. These sensors do not require additional mechanical or electrical compensations and are suitable for high-temperature applications. With 0.035% FS/K, the sensors exhibit remarkably high thermal stability at operating temperatures up to 500 °C. This is primarily attributed to the advantageous sensor design, enabling the production of an all-in-silicon sensor. This work focuses on advancing the design and manufacturing processes of these sensors to enable precise absolute measurements. A key innovation is the integration of a self-developed, advanced TSV technology. This integration required significant process optimization to seamlessly incorporate TSVs into the components, ensuring high reliability and performance while maintaining compatibility with the established manufacturing workflow.

**Figure 2:**
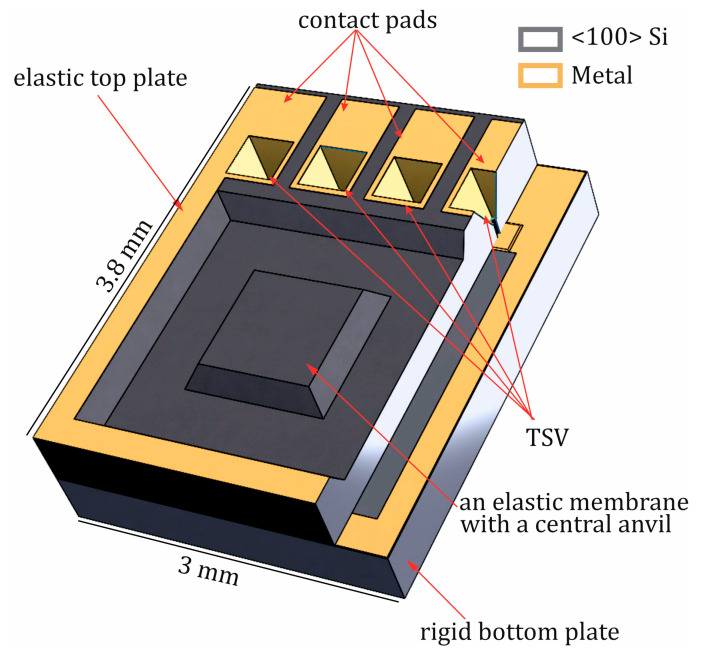
Three-dimensional model of the design concept with a transparent, perforated part of the top plate.

The sensors consist of two components, a bottom and a top plate, with single-crystal silicon being preferred due to its excellent thermal, mechanical, and elastic properties. The monocrystalline material exhibits virtually no plastic deformation before fracture, which is highly advantageous for the fabrication of spring elements [43]. By utilizing silicon as a purely mechanical component, temperature-dependent semiconductor effects are avoided. To minimize thermal stresses caused by the different temperature coefficients of the materials, both sensor plates are made of silicon. The sensor layout essentially follows a quasi-monolithic design, thus enhancing mechanical and thermal stability. A 3D model of the design concept is depicted in Figure 2.

The sensors are designed as standard plate capacitors, and the capacitance value is determined by the following equation:

(1) $$C=\;\varepsilon_{0}\varepsilon_{r}\frac{A}{d}$$

where “*C*” represents the capacitance, “*A*” denotes the electrode area, “*d*” signifies the electrode separation, “*ε_r_*” stands for the relative permittivity, and “*ε*_0_” represents the vacuum permittivity. Assuming that the relative permittivity “*ε_r_*” remains constant, the capacitance value is directly proportional to the electrode area and inversely proportional to the distance between the electrodes. In other words, a larger electrode area and a smaller electrode spacing are advantageous for achieving a higher capacitance value and thus a greater signal swing. Therefore, the electrode distance is set to 5 µm, resulting in an increased sensor sensitivity. Due to the inverse proportionality of capacitance to electrode spacing, a 50% change in distance (2.5 µm) leads to a 100% change in capacitance value.

**Figure 3:**
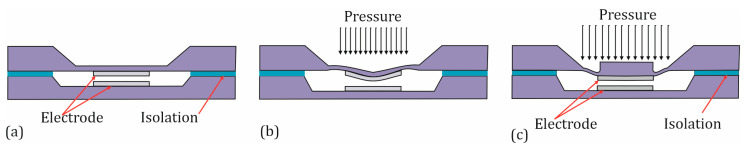
Cross-sectional side view of a standard capacitance sensor: (**a**) without load, (**b**) under load, and (**c**) with reinforcement of the membrane center under load.

Another important aspect of the specific configuration and design of the sensor is the deflection of the membrane, which directly affects linearity. Under idealized conditions where changes in electrode area and electrode spacing are proportional to the deflection of the sensor, a certain linearity could be expected. This mean that an accurate determination of the capacitance depends not only on the accuracy of the deflection of the membrane center but also on the accuracy of the deformed shape of the membrane [44]. Due to material behavior, for example, and the complex bending shapes of the membrane, the relationship between deflection and electrode geometry may be nonlinear, as illustrated in Figure 3b.

One way to increase linearity is to operate a capacitive sensor in contact mode [45,46]. In contact mode, the capacitance is nearly proportional to the contact area, which in turn exhibits good linearity with respect to the applied pressure across a wide pressure range. However, this linearity comes at the expense of a reduced sensitivity. Another method for enhancing linearity is also presented in [45] and involves reinforcing the center of the membrane, as shown in Figure 3c. By doing so, the center gains stiffness compared to the edge, and the electrode in the center undergoes minimal deformation under the applied load. This, in turn, increases the linearity of the capacitance–pressure characteristic. Our sensors are thus designed with a central anvil that directs forces or pressures over a defined area, ensuring improved linearity of the measurement signal. Additionally, the central anvil allows for the use of the sensors as force sensors without the need for further modifications.

**Figure 4:**
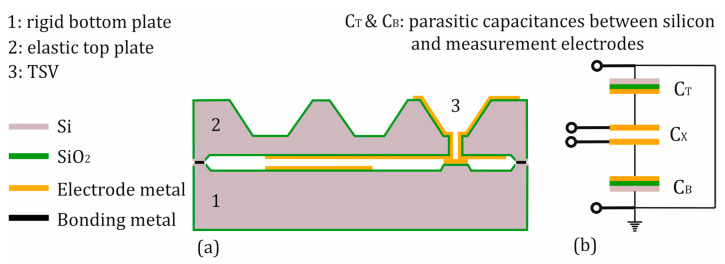
Cross-sectional side view (**a**) and electrical equivalent circuit (**b**) of the sensor.

The chip components are bonded together through eutectic bonding. Compared to other bonding methods such as silicon–silicon direct bonding, glass frit bonding, and anodic bonding, eutectic bonding does not require complex facilities, high voltage, or critical pretreatments [31,32]. Additionally, eutectic bonding exhibits a very high mechanical strength. For instance, the tensile strength is approximately 250 MPa for a Au-Si eutectic and around 140 MPa for an Al-Si eutectic [47,48]. Furthermore, eutectic bonding welds both sensor components together, mechanically and electrically connecting them. Additionally, the eutectic bonding process enables operation at higher temperatures of up to 500 °C for aluminum–silicon eutectic bonds [33] and up to 350 °C for gold–silicon shown in this publication. As a result, the capacitive measuring cell is surrounded by silicon material, which is a crucial aspect of this design. The silicon material forms a third electrode, which, along with the measuring electrodes, creates parasitic capacitances. This third electrode can be brought to a defined potential, allowing direct tapping of the measuring electrodes (floating mode) and avoiding the influence of parasitic capacitances on the measurement signal. Consequently, the output signal exhibits a much higher signal swing compared to conventional capacitive sensors. Furthermore, the silicon material forms a Faraday cage around the sensing electrodes, shielding the sensor against external electrostatic disturbances. Figure 4b depicts a schematic of the sensor’s equivalent circuit.

Unlike relative pressure sensors, absolute pressure sensors require a hermetically sealed cavity as a reference pressure chamber. The challenge lies in contacting the internal electrodes to the outside without compromising the hermetic integrity. To achieve this goal, we have developed an innovative method for manufacturing TSVs. Conventional TSVs are hermetically sealed by filling the via hole with a conductive material through electroplating, high-temperature deposition of poly-Si, or by using a conductive adhesive to ensure hermeticity. In contrast, the TSVs developed in this study are completely open, unlike conventional TSVs, and are not filled with conductive material. After bonding, they become completely sealed. This sealing is achieved by the thermocompression bonding of the vias to the opposing surface of the second substrate. This process will create an electrical contact between structures on both substrates and, at the same time, will hermetically seal the via contact surfaces. The manufacturing process will be explained in more detail in the next section.

By leaving the via holes open and avoiding complete filling with conductive material, the typical drawbacks of vias, especially in high-temperature applications, are avoided. This includes mechanical stresses that can be caused by different coefficients of thermal expansion of various materials, thereby reducing the risk of contact failure. Further potential issues such as corrosion, microcracks, and the formation of unwanted pn junctions are also circumvented. Moreover, this method significantly reduces manufacturing complexity since complex and elaborate processes such as grinding, polishing, electroplating, annealing, diffusion, high-temperature deposition, etc., are not required in this case.

### 3.2. Finite Element Simulation

**Figure 5:**
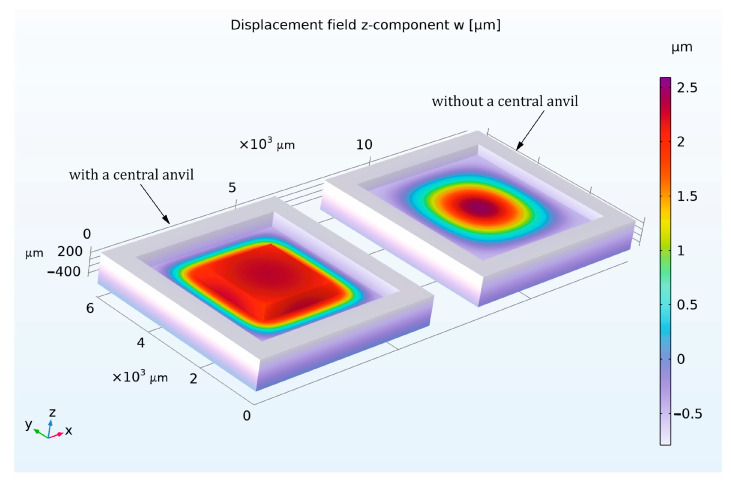
Comparison of the displacement field of the z-component (w) for the absolute sensor model with and without central anvil under 3 bar pressure and at 350 °C.

In order to achieve the optimal design parameters, a simulation was performed with COMSOL 6.2 and using the electromechanical physics interface. In the study, the same design described in the previous section was analyzed. As temperature changes are expected to have a greater effect on the performance of sensors with larger membrane areas and thinner membrane thicknesses, we focused on the planned largest sensor design (type F8) with the thinnest membrane in the simulation and later in the measurements. The material is monocrystalline silicon, where the anisotropic stiffness parameters of silicon are considered constant. The bottom of the base plate was fixed in the z-direction but could move in the plane. The measurement cavity is hermetically sealed, and the capacitance between the lower and upper electrodes is determined directly by integrating the charge distribution at a 1 V potential difference. The resulting electromechanical forces can be neglected. The electrode spacing is 5 µm, and the sensor dimensions are 6 mm × 6 mm. The effects of thermal expansion at 350 °C on the performance of the sensor are also considered. The study was analyzed under different ambient pressures. Figure 5 shows the deformation of the sensor membrane in the z-direction under an ambient pressure of 3 bar and a temperature of 350 °C.

In the model, a sensor with an anvil and 85 µm diaphragm thickness was compared with a sensor without an anvil and 160 µm diaphragm thickness. The diaphragm thickness was purposely chosen differently so that both sensors have approximately the same maximum measuring range of 2.55 bar. The maximum measuring range is defined by the 100% change in capacitance. As explained in the previous section, reinforcing the center of the membrane with an anvil results in the center increasing in stiffness compared to the edge and the electrode in the center being only minimally deformed under the applied pressure. Figure 5 illustrates this result and shows that the deflection of the membrane electrode with an anvil is more homogeneous and uniform over the entire electrode surface than with the sensor without an anvil.

**Figure 6:**
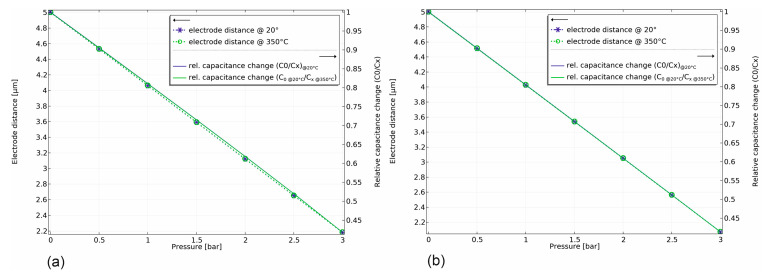
Performance of the sensor model at a total pressure of 3 bar and at 20 °C and 350 °C: (**a**) sensor without central anvil and (**b**) with central anvil.

The sensor performance shows that the 100% capacitance change is achieved at a displacement of 2.503 µm (half of the electrode distance) for the sensor with an anvil and 2.566 µm without an anvil. This in turn confirms the improved linearity of the capacitance–pressure characteristic, as shown in Figure 6.

However, with a small difference, the model shows exceptional thermal stability at 350 °C in both cases, with a thermal drift of only 0.00027% FS/K and 0.00024% FS/K under full load for the sensor without a central anvil and with a central anvil. This is expected as only a very small homogeneous thermal expansion of the whole sensor leads to a temperature dependence.

**Figure 7:**
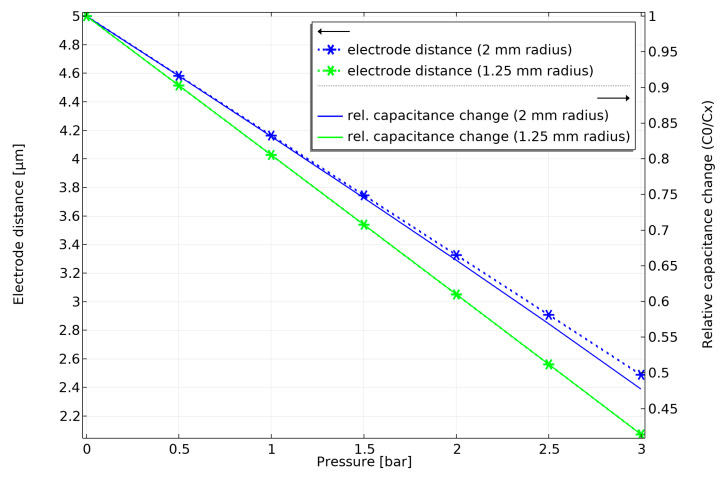
Comparison of sensor performance with an electrode radius of 1.25 mm and 2 mm.

Since a relatively uniform membrane displacement occurs beneath the anvil, the electrode size also plays a role in linearity. An electrode that is too large would increase the signal amplitude and thereby enhance sensitivity, but it would also degrade linearity. This is demonstrated in Figure 7. The electrode does not exceed the dimensions of the stiff anvil for a low electrode radius of 1.25 mm. Therefore, the sensor behaves like a perfect plate capacitor with a perfectly linear characteristic of inverse capacitance. Increasing the sensitivity by increasing the electrode radius to 2 mm leads to the incorporation of the more warped outer membrane region into the capacitor area, resulting in a deviation from perfect plate capacitor behavior. This reduces the linearity of the inverse capacitance by a small amount. Depending on the requirements of the sensor application, different tradeoffs between linearity and sensitivity can simply be adjusted by the electrode dimensions.

### 3.3. Manufacturing

**Figure 8:**
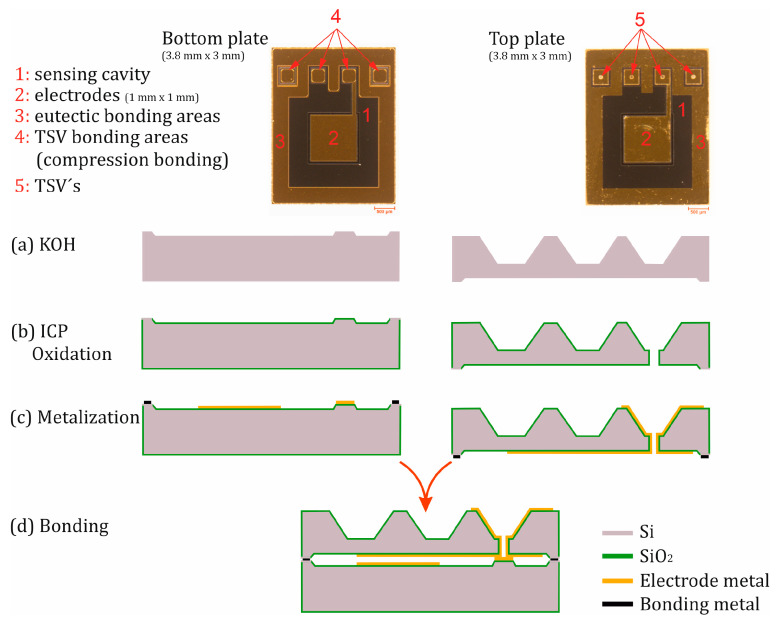
Top view of the front sides of the base and top plate with the schematic cross-sectional view of the clean-room process flow.

The production of the sensors relies on standard and industrial technologies for microelectromechanical systems (MEMSs) using monocrystalline <100>-silicon. Figure 8 illustrates a simplified representation of the process in a cleanroom.

**Figure 9:**
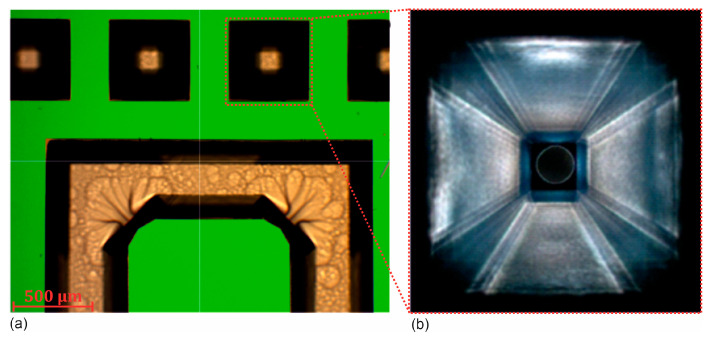
Top view of the backside of the top plate (**a**) after the KOH etch process and (**b**) of a TSV after opening the TSV hole by dry etching.

For the production of the absolute pressure sensor, two silicon wafers are processed in parallel to form the bottom plate and the top plate. The manufacturing process in Figure 8a begins in the etching bath with a two-stage KOH etching process. In the first step, the TSV holes and the elastic membrane with a central anvil on the backside of the top plate are etched (Figure 9a), and in the second step, a cavity for the capacitive sensor cell is structured on the front side of the substrates. This creates a sensor membrane with improved force coupling through the central anvil, which ensures better signal linearity. Next, the front side of the top plate is etched using a dry etching process (Inductively Coupled Plasma (ICP) etching) to open the TSV holes, before depositing and structuring an insulating layer of silicon oxide (SiO_2_) (Figure 9b).

In process step (c), the front side of the bottom plate and both sides of the top plate are metalized and structured. Spin coating in the lithography step of structures with a high aspect ratio like the back side of the top wafer is generally a challenge. The open TSVs also increase the complexity. To overcome this, the metallization of the front side of the top wafer was first performed by sputtering followed by a spin coating of the positive resist (AZ 1518 Microchemicals GmbH, Ulm, Germany), lithography, and wet-chemical metal etching. In a second step, the backside was treated with a bi-layer lift-off process (LOR 5A resist by micro resist technology GmbH, Berlin, Germany, and AZ 1518 Microchemicals GmbH, Ulm, Germany), followed by sputter metallization. This method enabled the successful metallization of the TSVs. This fabrication step led to some defects due to improper resist coating, which did not affect functionality. Nevertheless, resist deposition will be conducted by spray coating as soon as the equipment is available in our lab. Since the eutectic bonding occurs at the edges of the sensor cell, only the eutectic metal (Au) without oxides and adhesion layers is deposited and structured in this area. Therefore, the metallization step is carried out in two stages. Firstly, the electrodes, conductive paths, and TSV contact areas are coated with a suitable high-temperature adhesion and diffusion barrier layer, such as titanium–tungsten. In the second step, the eutectic metal is deposited. During the metallization of the front and back sides of the top plate, the insulated side wall of the TSVs is also metalized. Thus, in this step, an insulated capacitive sensor electrode, the insulated bonding area of the TSVs, and a metalized chip edge without insulation are structured. Finally, the top plate is aligned with the bottom plate, and they are bonded together by eutectic bonding (Figure 8d). Eutectic bonding has the advantage of fusing the bottom and top chips together, thus electrically and mechanically connecting them. As a result, the capacitive sensing cell is surrounded by silicon material, forming a Faraday cage around the sensing electrodes. With that, the sensor is shielded against external electrostatic disturbances. Another advantage of eutectic bonding is an operating temperature of up to 350 °C when Au is used as the eutectic metal. To increase the operating temperature to up to 500 °C, aluminum is used instead of gold. The electrodes are routed via a conductive path to the contact areas of the TSVs. Additionally, the TSV contact areas on the inner sides of both chips are simultaneously sealed during the bonding process. The pressure and temperature required for eutectic bonding bring the surfaces of the metal layers at the TSV contact areas of the bottom and top plates into direct contact and tightly compress them. This results in a solid mechanical connection known as a compression bond. When using gold, the layers are additionally welded together through the diffusion of gold atoms between the layers. This results in a metallurgical connection at the atomic level known as a diffusion bond [49]. Therefore, the hermetic sealing of the sensor is achieved through compression bonding or diffusion bonding at the TSV contact areas and through eutectic bonding at the outer edges of the sensing cells. The eutectic connection also ensures that the contact pressure at the TSV contact surface is maintained after solidification, providing additional strength. Furthermore, this combination of bonding techniques enables the production of sensors with high precision, reliability, and a high degree of sealing.

**Figure 10:**
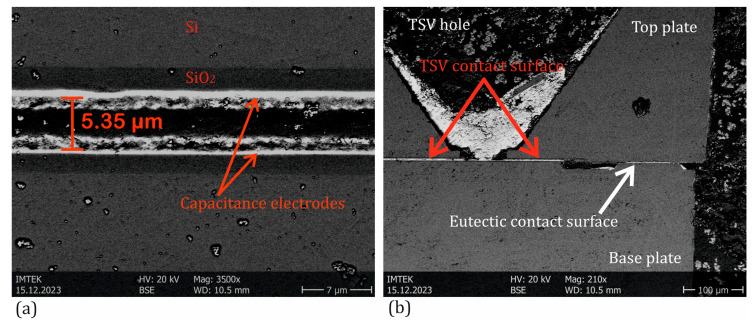
Microscopic cross-sectional image of a manufactured absolute pressure sensor. (**a**) The electrode distance. (**b**) A TSV, the TSV bonding area, and the eutectic bonding area.

The resulting gap between the capacitive electrodes after bonding is approximately 5 µm (Figure 10). This ensures a virtually displacement-free force measurement and increases the sensor’s sensitivity, as the capacitance is inversely proportional to the distance. A capacitance change of over 100% can be expected at a full-scale (FS) value. Figure 10 illustrates the resulting electrode gap as well as the TSV hole with the eutectic region of an absolute sensor after the bonding process, as shown by SEM investigation.

The bonding process described above was carried out in a specially designed oven for this application. During bonding, the sensors were placed in a vacuum chamber positioned between two 4-inch quartz discs. Halogen lamps were mounted above and below the quartz discs to heat the bonding substrates. The upper disc applied pressure to the sensors through the vacuum at a pressure of 1 bar, equivalent to a total force of 785 N. Depending on the chip size, 2 to 4 sensors were bonded together in one batch, resulting in a bonding force of 195 N to 390 N per sensor. The halogen lamps consumed approximately 50 W in total to achieve a bonding temperature of over 400 °C. The initial bonding attempts with a direct setting of 50 W (rapid heating) and a total process duration of 4 min resulted in sensors that were eutectically bonded but not hermetically sealed. They functioned practically as relative sensors. The corresponding parameter search was time-consuming since the sensors need to be prepared on a ceramic PCB through wire bonding after each bonding process to check their tightness in a pressure chamber. Eventually, we were able to find suitable parameters with the simple bonding oven. The best results were achieved by slow heating in three stages (25 W for 30 s, 40 W for 1 min, and 50 W for 4 min), followed by maintaining the process temperature for 4 min and rapid cooling. The total process duration was 8 min.

**Figure 11:**
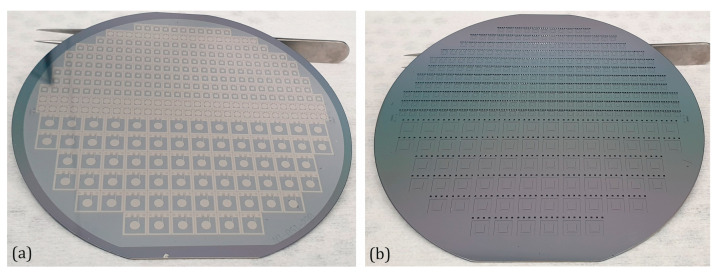
Top view of a structured top wafer of the absolute pressure sensors with membrane anvil. (**a**) Front side after metallization step and (**b**) back side after oxidation step.

**Figure 12:**
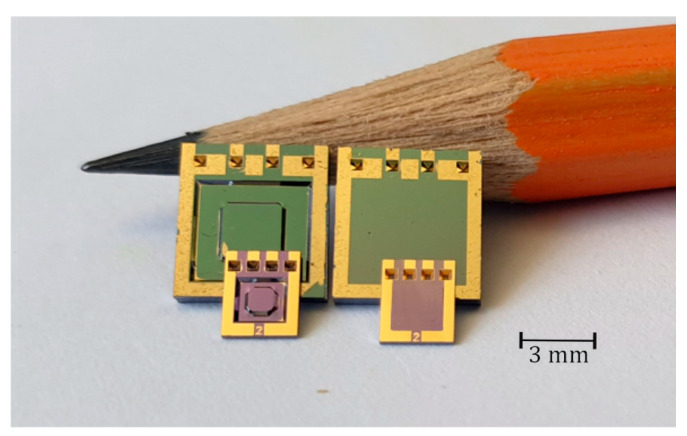
Top view of different sensor sizes manufactured on the same wafer pair. Type F8 (chip dimension 6.8 mm × 6 mm × 0.9 mm) and Type F2 (chip dimension 3.8 mm × 3 mm × 0.9 mm), each with and without anvil.

With the described design and manufacturing method, two different sensor sizes were produced. A significant advantage of this manufacturing process is that it is independent of the design parameters. This means that in a batch, different sensor designs for various measurement ranges can be manufactured. To illustrate the flexibility of the manufacturing process, the two different sensor dimensions with different sensor designs were produced on the same wafer pair, as shown in Figure 11 and Figure 12. Each wafer contains two sensor sizes: the top side of the wafer has sensors with dimensions of 3 mm × 3.8 mm, while the larger sensors, measuring 6 mm × 6.8 mm, are located on the bottom side. The sensor designs differ in the shape of the electrode (round or square) and the membrane structure (with or without anvil). In addition to sensor dimensions, the measurement range can also be adjusted by the membrane thickness.

## 4. Results

**Figure 13:**
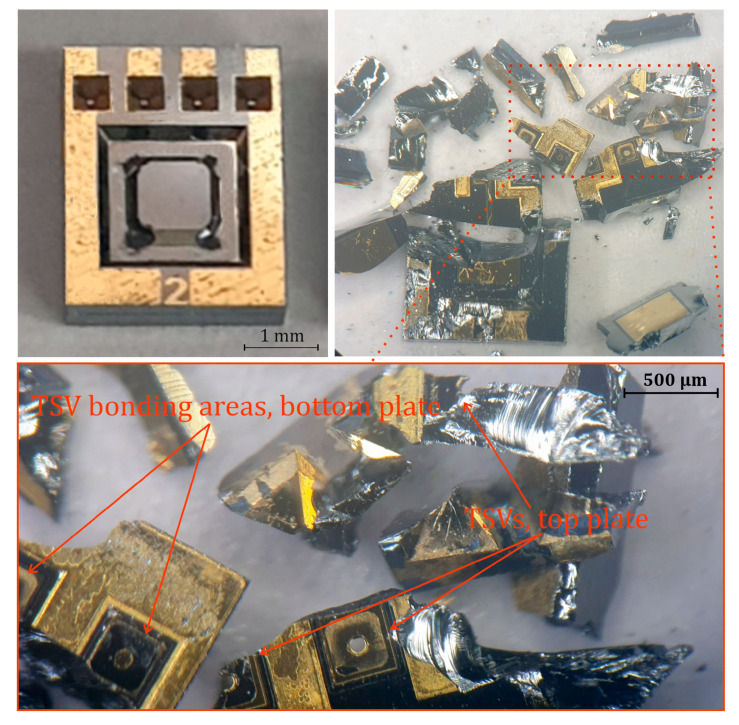
Light microscope images of the scalpel test results of a type F2 absolute sensor.

To analyze the bonding results and mechanical properties, the chips were fractured using a scalpel and force and subsequently examined under a light microscope. The objective was to separate the sample along the bond interface in order to closely evaluate the bond quality. Figure 13 illustrates the test results of a type F2 absolute sensor with a membrane and anvil. The test clearly demonstrates the diffusion of Si into Au and the formation of a eutectic phase between the two sensor plates. Complete separation of the components without breaking them was not possible. The fractures were mainly located in the silicon bulk and not on the surface of the bonding area, which indicates a strong and robust bond. The TSVs, where the compression bonding was very successful, can also be clearly recognized. The metallization layer of the TSVs on the top side cracked and remained adhered to the TSV bonding areas on the bottom side, as shown in Figure 13.

To support these results, the shear strength was investigated in relation to the bonding parameters described in the previous section. For the shear strength test of two chips bonded using rapid heating and two bonded using slow heating, the shear testing machine Sigma Condor from XYZTEC with a force capacity of 2000 N and a shear speed of 0.05 mm/s was used. Unlike the chips bonded with rapid heating, the shear strength reached up to 60 MPa for those bonded with slower heating. Table 2 compares the results between the four samples.

**Figure 14:**
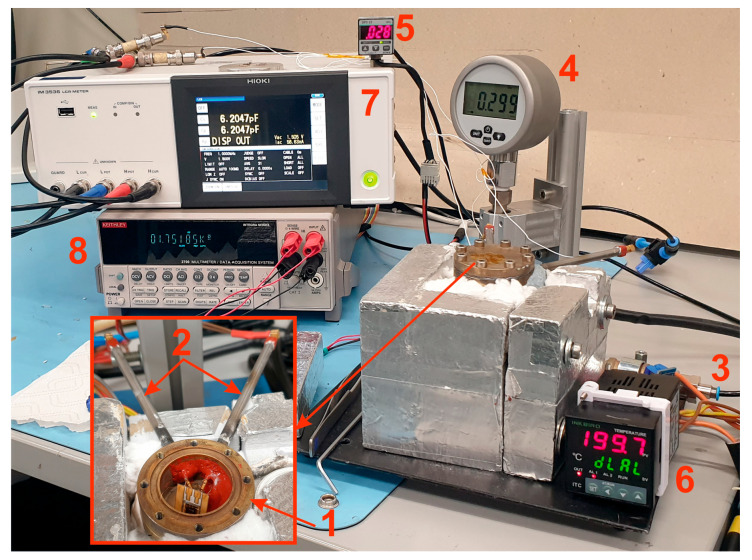
Test setup with (1) pressure chamber with the sensor inside, (2) coaxial feedthrough contacts, (3) pressure inlet, (4) Digi-04 DM-04 pressure sensor, (5) SUNX DP2-22 pressure sensor, (6) temperature controller, (7) LCR meter, and (8) Pt1000 readout.

For the initial characterization, a heated brass pressure chamber was fabricated (Figure 14). The chamber is heated by a resistance heater. The ITC-100VL controller from INKBIRD (Shenzhen, China), together with a Pt-1000 temperature sensor (Kleinostheim, Germany) and a thermocouple, ensures precise temperature control. Two coaxial feedthroughs for sensor connection are integrated into the chamber. The lid, also made of brass, is screwed onto the pressure chamber with a 500 µm thick graphite foil. The pressure is set using a mechanical pressure regulator and then measured by two pressure gauges, the Digi-04 DM-04 (Bräunlingen, Germany) with a 2.5 bar maximum pressure range and the Panasonic SUNX DP2 22 (Kadoma, Japan) with a 10 bar maximum pressure range. The Digi-04 DM-04 has a resolution and accuracy of 10 mbar and 0.4%, respectively, while the SUNX DP2-22 has a resolution of 10 mbar and a repeatability of 0.2% FS. The sensor is attached to a ceramic printed circuit board (PCB) at two points using high-temperature resistant silicone and is wire-bonded to the PCB. The PCB is wired using electric welding and connected to the coaxial connector of the pressure chamber. The sensor capacitance is measured using an LCR meter (HIOKI IM3536, Nagano, Japan) operating at a frequency of 10 kHz, a voltage amplitude of 1.5 V, and a resolution of 0.0001 pF.

The measurements were carried out with an F8 membrane–anvil sensor with a diaphragm area of nearly 25 mm^2^ and a thickness of 85 µm, making it the largest and thinnest in the series. The overall sensor dimensions are 6 mm × 6.8 mm × 0.9 mm.

**Figure 15:**
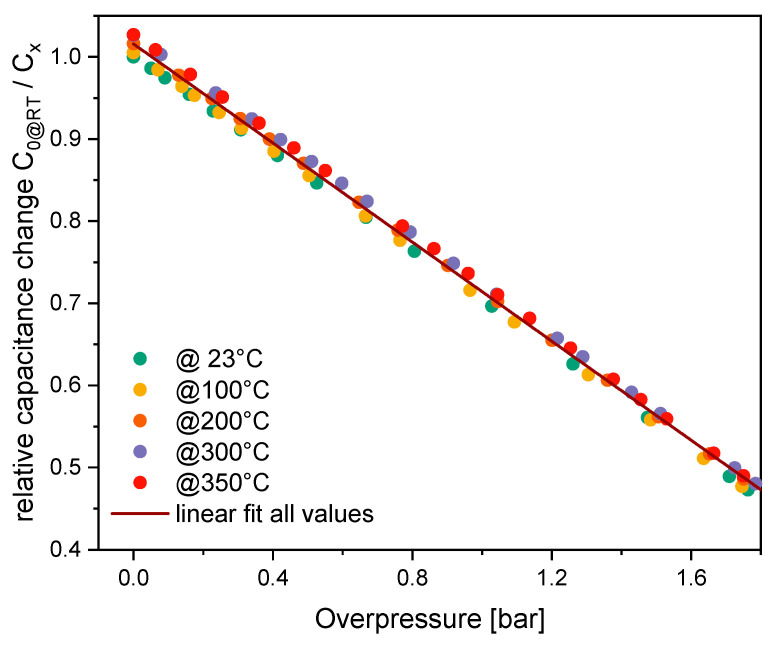
Relative capacitance change vs. load up to 350 °C of an absolute F8-type sensor.

Despite the simple design and potential sources of error caused by heating of the setup components, the measurement results remain consistent over extended periods, delivering promising outcomes. The inverse capacitance vs. pressure curves were recorded at 23 °C, 100 °C, 200 °C, 300 °C, and 350 °C. The results demonstrate a high degree of linearity, with 99.993% at room temperature (RT) and 99.994% at 350 °C, over a range exceeding 100% of the initial capacitance change (Figure 15). The initial capacitance (C_0@RT_) is 11.165 pF, reaching 23.614 pF at 2.763 bar FS at RT, resulting in a sensor sensitivity of 3.342 pF/bar. The temperature coefficients are −0.0066%/K under no load and −0.0164% FS/K at full load at 350 °C.

**Figure 16:**
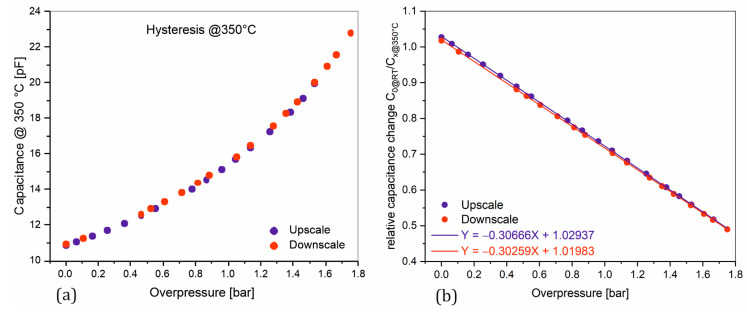
Hysteresis measurement at 350 °C. Upscale and downscale of (**a**) the capacitance change vs. load and (**b**) the relative capacitance change vs. load.

Figure 16a illustrates the forward and backward measurements of the sensor at 350 °C in order to determine its hysteresis. Hysteresis represents the difference between the forward and backward measurements, typically observed at a specific pressure point. It is generally calculated using the following equation:

(2) $$U_{hysteresis}=Max\left|x_{up}-x_{down}\right|$$

where the following definitions hold:

- x_up_ denotes the sensor’s response during the forward (increasing pressure) measurement;
- x_down_ represents the sensor’s response during the backward (decreasing pressure) measurement.

In this context, hysteresis provides a measure of the lag or deviation between the increasing and decreasing pressure cycles, which is a key parameter in evaluating the performance and accuracy of pressure sensors under thermal stress, such as at elevated temperatures like 350 °C. The greater the hysteresis, the larger the error introduced in pressure readings due to this effect, which must be minimized for precise sensor applications.

Since the applied overpressure cannot be set exactly, meaning that the forward and backward measurements are not taken at identical points, the hysteresis is analyzed through the linear behavior and expressed as a function of overpressure (Figure 16b) by directly subtracting the two linear equations:

(3) $${U(x)}_{hysteresis}={F(x)}_{up}-{F(x)}_{down}$$

Therefore, the hysteresis at a given overpressure, in relation to C_0_/C_x_, is described by the following equation:

(4) $${U(x)}_{hysteresis}=-0.00407\cdot x+0.00954$$

Given that the slope term is negative, the hysteresis decreases slightly with increasing overpressure. As a result, the maximum hysteresis with respect to C_0_/C_x_ occurs at an overpressure of 0 bar and is equal to 0.00954.

**Figure 17:**
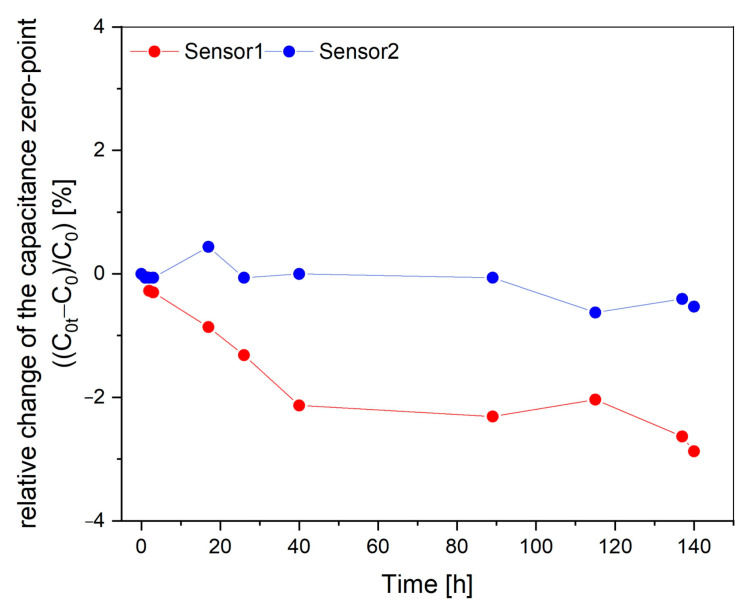
Relative change in zero-point capacitance of two absolute sensors annealed at 350 °C for 140 h under atmospheric pressure.

To evaluate the reliability and long-term stability of the sensor, particularly under extreme conditions such as high-temperature environments, two sensors were annealed at 350 °C for 140 h under atmospheric pressure in a Heraeus furnace. The furnace was preheated to the target temperature before inserting the sensors. At specific intervals, as illustrated in Figure 17, the sensors were removed from the furnace and placed on a room-temperature aluminum block to measure their zero-point capacitance (no load). After the measurement, the sensors were immediately returned to the furnace.

This process involved repeated rapid cooling and reheating, subjecting the sensors to significant mechanical and thermal stress. Despite these rigorous conditions, the measurements yielded promising results. The same LCR meter and parameter settings from the previous measurement were used for consistency. The relative change in the zero-point capacitance after 140 h at 350 °C was less than −3% for sensor 1 and even far below −1% for sensor 2, demonstrating the sensor’s robust performance and thermal stability. The capacitance decreased slightly over time for both sensors.

## 5. Discussion

The manufacturing process presented in this study is based on industrial MEMS technology, enabling the production of various sensor designs that cover a wide range of pressure measurements. The measurement range can be adjusted by modifying the sensor dimensions and membrane thickness. The complete structural material used is monocrystalline silicon, which minimizes thermal stress in the sensor design. Additionally, temperature-dependent semiconductor effects are avoided since silicon functions purely as a mechanical material rather than a semiconductor. Consequently, temperature changes only influence the thermal expansion coefficient and the elastic modulus, both of which have negligible effects on silicon. As a result, temperature compensation is generally unnecessary, as demonstrated by the sensor’s stable signal and pressure sensitivity across different temperature ranges.

Moreover, the flexibility of the eutectic bonding step in the manufacturing process enables the sensors to operate at temperatures up to 350 °C. By using a eutectic aluminum–silicon bonding technique, an operating temperature of up to 500 °C would even be possible, as demonstrated in our previous work on relative sensor developments [33]. The capacitive sensor electrodes are shielded by a Faraday cage formed from the surrounding silicon itself, providing excellent protection against external electrostatic interference without any additional effort.

An essential aspect of the fabrication process is the innovative design of TSVs, which offer several advantages. Although the TSVs are continuous openings through the substrate, they form a hermetically sealed surface when bonded with the second substrate. This sealing is achieved through Au-Au diffusion bonding (thermocompression) and Au-Si eutectic bonding, ensuring that the contact pressure on the TSV surfaces remains stable after bonding. The strong bond between the TSV contact areas (Figure 13 and Table 2) enhances the mechanical and thermal stability of the sensor, even at elevated operating temperatures. Furthermore, the unfilled TSV openings eliminate issues related to mechanical stress due to thermal expansion.

In general, the manufacturing process with such a high aspect ratio is a challenge, particularly in lithography. In our process, the double-sided patterning of the top wafer, especially after opening the TSVs, further increased the complexity. By using bi-layer lithography, we were able to solve this problem, but due to the spin coating process, there were some defects on the wafer. This step could be further optimized by using spray coating. The long-term experiment with harsh temperature shocks shown in Figure 17 shows a slight drift of the zero-load capacitance over time towards lower capacitance values. The number of sensors as well as the duration of the experiment do not allow for a final evaluation of this trend. A small leakage of the cavity seal might be the reason, which would require further optimization of the TSV process. On the other hand, this drift seems to saturate far below the value expected for a total pressure loss; therefore, effects other than leakage should also considered in further research. The bonding was carried out using a custom-built oven with halogen lamps for heating. The bonding force was generated by the pressure difference between the vacuum chamber and atmospheric pressure. However, the design of the oven limited our ability to precisely control or adjust the bonding force, impacting the bond strength. Bonding process parameters like temperature and bonding force as well as the width of the sealing frame might further be optimized to increase the stability of the sensor signal.

The flexibility of the TSV fabrication process also allows for the placement of TSVs on the backside of the base plate with minimal additional effort. This permits electrical isolation of the contact pads from the sensing medium when necessary. This adaptability, coupled with the use of dry etching instead of anisotropic wet etching, enables the production of sensors with compact dimensions and the customization of membrane structures to suit various load conditions. Furthermore, silicon structuring techniques are well established, standardized, and straightforward, making the manufacturing process more efficient.

The optimum design parameters were analyzed using finite element simulations. The results illustrate the flexibility of the design and the potential for easy application-specific customization. The sensor dimensions, the membrane thickness, the anvil, and the electrode size allow the sensors to be adapted to different applications.

Despite the possible sources of error associated with the simple measurement setup, the measurements showed stable signals at different temperatures and an impressive linearity of 99.994% with a thermal drift of only −0.0164% FS/K at full load and 350 °C. This very low thermal drift is still much higher than 0.00024% FS/K expected from finite element simulation, indicating that not only the homogeneous thermal expansion determines this drift. The reason for this deviation cannot yet be conclusively determined with the current knowledge. The measured thermal drift might be an artifact originating from the thermal drift of the measurement setup or might be caused by stresses from the thermal mismatch of the eutectic bonding layer to the bulk silicon, which were not considered in the simulation due to the lack of experimental data on the properties of the bonding layer.

Nevertheless, the sensors show up to 10 times better thermal stability and significant improvement in linearity compared to the other published work, as shown in Table 3.

The maximum measuring range of 2.76 bar, achieved at a 100% change in capacitance, aligns very well with the simulated value of 2.55 bar. The small discrepancy of approximately 200 mbar is likely due to the KOH etching error of the membrane. Simulations indicated that a mere 5 µm variation in membrane thickness for the F8 membrane anvil sensor results in a total change in the measuring range of 300 mbar. Additionally, the significant range of capacitance change, exceeding 100% of the no-load capacitance, guarantees the high sensitivity of the sensors, achieving a respectable sensitivity of 34 fF/kPa.

## 6. Conclusions and Outlook

The presented capacitive absolute pressure sensors are fabricated using industrial MEMS technology, employing monocrystalline silicon exclusively as a mechanical material, not as a semiconductor. This choice minimizes thermal stresses and negates the need for temperature compensation, as demonstrated by the sensors’ stable signals and consistent pressure sensitivity across varying temperatures. The sensing capacitance is surrounded by silicon, forming a Faraday cage that effectively shields the sensor from external interference. By optimizing sensor dimensions and membrane thickness, the sensors can be adapted for a wide range of measurement requirements.

The integration of a novel TSV design, combined with advanced eutectic and diffusion bonding techniques, ensures the sensors’ mechanical and thermal stability, even at elevated operating temperatures. This flexibility in design, supported by established silicon structuring methods, enables the production of reliable sensors with customizable dimensions. Additionally, the self-packaging capability of the TSV design allows for direct integration into various applications, thus further enhancing the sensors’ versatility.

The sensors demonstrated outstanding performance, with a high linearity of 99.994%, a minimal thermal drift of −0.0164% FS/K at 350 °C, and a notable sensitivity of 34 fF/kPa. These promising results suggest that the developed sensors have significant potential for further advancement and application in a wide range of high-performance, demanding environments.

Future work will focus on optimizing the fabrication process to overcome the current limitations, particularly in TSV metallization and wafer bonding. With the newly acquired spray coater, challenges associated with spin coating over high-aspect-ratio structures will be addressed, enabling more uniform and defect-free structuring. Additionally, the integration of an advanced wafer–wafer bonding system will allow for precise control of bonding force and temperature, significantly improving bond strength and hermeticity. These advancements will enable more detailed investigations into long-term device reliability, including extended thermal cycling and endurance tests with an increased sample size.

## Figures and Tables

**Table 1:** The main advantages and disadvantages of the most common pressure and force sensor technologies [33].

Sensor Principle	Optical	Piezoelectric	Piezoresistive	Capacitive
Advantages	high accuracy high resolution insensitive to electromagnetic interference high operating temperature	ease of integration high operating temperature wide measuring range high resolution	low cost simple signal processing circuit miniaturized good linearity	high sensitivity high accuracy low power consumption high shock resistance
Disadvantages	temperature-sensitive complex signal processing circuit	complex electronic interface only dynamic measurement expensive	high power consumption sensitive to temperature and mechanical stress	susceptible to parasitic and stray capacitance complex fabrication complex signal processing circuit

**Table 2:** Comparison of shear strength results for chips bonded with rapid and slow heating.

No.	Bonding Heating Art	Duration [min]	Shear Strength [MPa]
1	rapid	4	42
2	rapid	4	44
3	slow	8	58
4	slow	8	60

**Table 3:** Comparison of performance with other research work on the development of pressure sensors.

Reference	[10]	[50]	[51]	[2]	[3]	[52]	F8-Type
Sensor principle	optical	piezoelectric	piezoresistive	piezoresistive	capacitive	capacitive	capacitive
Thermal stability	15.7 pm/°C	≤5%	0.15% FS	0.05% FS/K	1% **	0.046%/K	−0.016% FS/K
Max temperature	1000 °C	650 °C	350 °C	200 °C	600 °C	574 °C	350 °C
Nonlinearity	-	≤1%	0.17% FS *	0.18% FS	6%	2.4%	0.006% FS

* Combined with hysteresis, nonlinearity, and repeatability. ** Zero-point drift.

## Data Availability

The original data presented in the study are openly available at https://bwsyncandshare.kit.edu/s/n3FxttF2RSjWQbi (accessed on 19 January 2025).
